# Fluid flow exposure promotes epithelial-to-mesenchymal transition and adhesion of breast cancer cells to endothelial cells

**DOI:** 10.1186/s13058-021-01473-0

**Published:** 2021-10-12

**Authors:** Kenneth F. Fuh, Robert D. Shepherd, Jessica S. Withell, Brayden K. Kooistra, Kristina D. Rinker

**Affiliations:** 1 Cellular and Molecular Bioengineering Research Lab, University of Calgary, Calgary, AB Canada; 2 Department of Chemical and Petroleum Engineering, University of Calgary, 2500 University Drive NW, Calgary, AB T2N 1N4 Canada; 3 Centre for Bioengineering Research and Education, University of Calgary, Calgary, AB Canada; 4 Department of Physiology and Pharmacology, University of Calgary, Calgary, AB Canada; 5 Charbonneau Cancer Institute, University of Calgary, Calgary, AB Canada

**Keywords:** Breast cancer, Fluid flow, Metastasis, Epithelial-to-mesenchymal transition, Adhesion

## Abstract

**Background:**

Mechanical interactions between tumor cells and microenvironments are frequent phenomena during breast cancer progression, however, it is not well understood how these interactions affect Epithelial-to-Mesenchymal Transition (EMT). EMT is associated with the progression of most carcinomas through induction of new transcriptional programs within affected epithelial cells, resulting in cells becoming more motile and adhesive to endothelial cells.

**Methods:**

MDA-MB-231, SK-BR-3, BT-474, and MCF-7 cells and normal Human Mammary Epithelial Cells (HMECs) were exposed to fluid flow in a parallel-plate bioreactor system. Changes in expression were quantified using microarrays, qPCR, immunocytochemistry, and western blots. Gene–gene interactions were elucidated using network analysis, and key modified genes were examined in clinical datasets. Potential involvement of Smads was investigated using siRNA knockdown studies. Finally, the ability of flow-stimulated and unstimulated cancer cells to adhere to an endothelial monolayer, migrate and invade membrane pores was evaluated in flow and static adhesion experiments.

**Results:**

Fluid flow stimulation resulted in upregulation of EMT inducers and downregulation of repressors. Specifically, Vimentin and Snail were upregulated both at the gene and protein expression levels in flow stimulated HMECs and MDA-MB-231 cells, suggesting progression towards an EMT phenotype. Flow-stimulated SNAI2 was abrogated with Smad3 siRNA. Flow-induced overexpression of a panel of cell adhesion genes was also observed. Network analysis revealed genes involved in cell flow responses including FN1, PLAU, and ALCAM. When evaluated in clinical datasets, overexpression of FN1, PLAU, and ALCAM was observed in patients with different subtypes of breast cancer. We also observed increased adhesion, migration and invasion of flow-stimulated breast cancer cells compared to unstimulated controls.

**Conclusions:**

This study shows that fluid forces on the order of 1 Pa promote EMT and adhesion of breast cancer cells to an endothelial monolayer and identified biomarkers were distinctly expressed in patient populations. A better understanding of how biophysical forces such as shear stress affect cellular processes involved in metastatic progression of breast cancer is important for identifying new molecular markers for disease progression, and for predicting metastatic risk.

**Supplementary Information:**

The online version contains supplementary material available at 10.1186/s13058-021-01473-0.

## Introduction

Breast cancer is a heterogeneous disease that progresses from oncogenic transformation in local epithelial cells to formation of tumors at distant organ sites [1, 2]. Early diagnosis and stratification is important for determining patient treatment options and improving outcomes, thus promoting precision medicine [3, 4]. All steps in the metastatic cascade involve mechanical interactions between tumor cells and the different dynamic microenvironments they encounter, including exposure to fluid flow [5–7]. Tumor cells encounter two types of fluid flow: interstitial flow in the tumor microenvironment and fluid flow in the vascular or lymph microenvironment [6, 8].

In vitro models that mimic interstitial fluid flow patterns have been used to show flow-induced changes in several cell types and implications for disease progression and treatment have been reviewed by Munson and Shieh [9]. For example, interstitial fluid flow induced invasion of HER2-expressing breast cancer cells [10], potentiated mobility in metastatic esophageal cancer cells [11], regulated invasiveness in glioma cells [11, 12], and modulated receptor-mediated apoptosis in lung cancer cells [13]. Also, exposure of ovarian cancer cells to fluid flow in a microfluidic platform induced Epithelial-to-Mesenchymal Transition (EMT) and generated a more aggressive and motile phenotype in 3D ovarian micronodules [14]. Although fluid flow is known to significantly affect cancer cell’s behavior, not much information is available on how forces of the magnitude of those experienced in the vascular microenvironment affect cellular events during cancer progression. Forces of such magnitudes (0.1–1 Pa) have been shown to affect cell phenotypes by activating signaling pathways and inducing transcription factors, some of which are involved in EMT [15, 16].

The process of EMT causes significant alterations in the adhesive and mechanical properties of cells, facilitating tumor cell detachment from the primary tumor [7, 17, 18]. EMT is characterized by loss of the structured epithelial morphology and gain of the slender morphology of mesenchymal cells [19, 20]. This is accompanied by downregulation of epithelial genes such as CDH1 and KRT19, and upregulation of mesenchymal genes including FN1, VIM, CDH2, SNAI1, SNAI2, and MMPs [17, 20, 21]. Resulting morphological and behavioral changes cause mesenchymal-like characteristics, including cells becoming more motile and invasive [22–24]. The TGF-β pathway is well known for its involvement in EMT in cancer cells. Knowledge of how mechanical forces such as fluid flow promote EMT and metastatic events would allow for identification of key genes that can be used to facilitate early diagnosis and stratification of breast cancer with the goal of better determining patient treatment options.

Once a tumor cell survives the biophysical forces posed by blood flow, it must adhere to the endothelial cells lining blood vessels at sites of secondary tumor formation, extravasate, and survive before initiating growth [25]. Extravasation is a multi-step process characterized by initial cell capture, tethering, rolling, firm adhesion, and subsequent transmigration through endothelial cells [26, 27]. Cancer cell adhesion requires expression of cognate ligands and receptors on cancer and endothelial cells, notably integrins [28, 29], collagens [29], selectins [30, 31], and Activated Leukocyte Cell Adhesion Molecule (ALCAM) [32–34]. Some of these adhesion molecules have been shown to play pivotal roles in cancer progression [30, 35]; however, no study has investigated how prior exposure of breast cancer cells to the shear stresses derived from blood flow affects expression of genes involved in facilitating subsequent adhesion to endothelial cells.

We hypothesized that stimulation of breast cancer cells to shear stress derived from fluid flow increases adhesion of breast cancer cells to endothelial cells. A bioreactor system consisting a parallel-plate flow chamber was used to expose breast cancer cells to moderate levels of fluid flow, like those found in blood vessels. Gene expression analysis was used to evaluate flow-induced changes in breast cancer cells. The ability of flow-stimulated and unstimulated breast cancer cells to adhere to an endothelial monolayer in vitro was also evaluated. Network analysis was used to identify key interactions between genes involved in promoting EMT and cell adhesion. Significant findings were analyzed in a clinical dataset consisting of gene expression data for more than 600 breast cancer patients. The prognostic value of key differentially expressed genes was evaluated by comparing relapse-free survival times in 2878 breast cancer patients.

## Materials and methods

### Cell culture and flow experiments

Human Mammary Epithelial Cells (HMECs) and BT-474 cells were respectively obtained from Lonza (Walkersville MD, USA) and ATCC (American Type Culture Collection, Manassas VA, USA) and cultured per recommendations. MCF-7 and SK-BR-3 cells were provided by Dr. Carrie Shemanko (Department of Biological Sciences, University of Calgary) while MDA-MB-231 cells were provided by Dr. Don Morris (Tom Baker Cancer Center, University of Calgary). MCF-7, SK-BR-3, and MDA-MB-231 cells were grown in Dulbecco's Modified Eagle's Medium (Life Technologies Inc., ON, Canada) supplemented with 10% fetal bovine serum, 1% L-Glutamine and 1% Penicillin Streptomycin. MCF-7, BT-474, MDA-MB-231, and SK-BR-3 cell lines are representative cell lines of luminal A, luminal B, basal, and HER2-enriched subtypes of breast cancer, respectively [36]. Disease-relevant differences between breast cancer subtypes have been described elsewhere [37–39].

For bioreactor culture, cell monolayers were cultured on glass plates pre-coated with 145 µg/mL Rat Tail collagen I (Life Technologies Inc., ON, Canada) and grown to confluence. Some slides were exposed to fluid flow by setting up the parallel-plate flow chamber as previously described [15], with the addition of pulse dampeners to create steady flow. Some glass plates were grown as static controls, and their culture media was replaced at the same time as flow was set up. The magnitude of shear stress on cell monolayers was calculated using the Navier–Stokes equation for a Newtonian fluid in parallel plate geometry as previously reported [15]. Cells were exposed to an average shear stress of 1 Pa for 20 h, and flow was provided by a Masterflex peristaltic pump and tubing (Cole Parmer, Montreal, QC, Canada). For each cell line, a minimum of three independent experiments were conducted for each condition.

### RNA extraction

Cells from flow experiments and static cultures were harvested as previously described [15]. Total RNA was isolated using the EZNA Total RNA Kit (Omega Bio-Tek Inc., Norcross GA, USA) as per the manufacturer’s instructions. Hydrated RNA samples were stored at –80 °C until analysis. RNA was assayed using the Quant-iT™ RiboGreen RNA Assay Kit (Life Technologies Inc., Burlington ON, Canada) and standard curves generated using a microplate reader.

### Real-time quantitative PCR

1 µg of RNA was converted to complementary DNA (cDNA) using the RT^2^ First Strand Kit (SA Biosciences, Mississauga ON, Canada) according to the manufacturer’s protocol. cDNA for each sample was loaded onto the RT^2^ Profiler PCR Arrays for Human Epithelial-to-Mesenchymal Transition (SA Biosciences, Mississauga ON, Canada) along with SYBR Green ROX qPCR Mastermix (SA Biosciences, Mississauga ON, Canada). Quantitative PCR was carried out on a ViiA 7 Real Time PCR System (Life Technologies, Foster City CA, USA) under the following conditions: 95 °C for 10 min and 40 cycles of 15 s at 95 °C and 60 s at 60 °C. Actin-beta (ACB), beta-2-microglobulin (B2M), and glutaraldehyde-3-phosphate dehydrogenase (GAPDH) were used as reference genes. Relative gene expression was calculated using the comparative cycle threshold method [40]. Three independent replicates were run for each condition and statistical significance for differential expression of genes between conditions was determined using the Student’s *t*-test.

For individual gene expression quantification by PCR, cDNA was synthesized using the qScript™ cDNA Synthesis Kit (Quanta Biosciences, Gaithersburg MD, USA) according to the manufacturer’s protocol. A total reaction volume of 20 µl was then prepared by adding 2 µl cDNA, 10 µl TaqMan® Fast Advanced Master Mix (Thermo Fisher Scientific, Ottawa, ON, Canada), and 8 µl mixture of primers, probes, and nuclease-free water. Quantitative PCR was carried out on a ViiA 7 Real Time PCR System (Life Technologies, Foster City CA, USA). PCR experiments were run in triplicates and B2M was used as the reference gene. The following cycling conditions were employed for target gene amplification: 50 °C for 2 min, then 95 °C for 20 s, followed by 40 cycles of 1 s at 95 °C and 20 s at 60 °C. Three independent replicates were run for each condition and relative gene expression was calculated using the comparative cycle threshold method [40]. Statistical significance for differential expression of genes between conditions was determined using the Student’s *t*-test.

### Protein extraction

Cytoplasmic/membrane protein fractions were obtained as described previously [16], with all solutions prechilled on ice and samples stored at –80 °C until analysis. Following this, nuclear proteins were harvested by first rinsing the plate twice with cold PBS to remove any remaining cytosolic protein. Cold-inhibited MES buffered saline [MBS; 25 mmol/l MES; 0.15 mol/l NaCl (pH 6.5); 4% complete protease inhibitor (Roche Applied Science, Laval, QC, Canada), and 1% halt phosphatase inhibitor cocktail (Thermo Fisher Scientific, Waltham, MA, USA)] were added to the attached nuclei. Nuclei were then removed from the glass by scraping with a cell scraper, collected, and stored on ice. A second volume of MBS was added, and the scraping and collection repeated. A final wash of the glass plates was performed with an additional MBS volume, and this was also added to the nuclei sample. A volume of 10% SDS was added to achieve a final SDS concentration of 0.1% in the samples, and the nuclei were extracted for 5 min at room temperature by vortexing. Following extraction, nuclear protein samples were concentrated to a final volume of < 100 µl using Amicon Ultra Centrifugal concentration tubes (Millipore, Billerica, MA, USA) and stored at –80 °C until analysis.

### Western blotting

Protein concentrations were determined using the BCA kit (Thermo Scientific-Pierce), and samples denatured, run on SDS-PAGE gels, and electroblotted as previously described [16]. Blot membranes were blocked for 1 h in ECL Advance Blocking Agent (GE Healthcare, Baie D'urfe, QC, Canada), and primary antibodies were applied at an appropriate dilution (1:1000–1:2000) in blocking agent and incubated overnight at 4 °C on a shaker. The following primary antibodies, obtained from Cell Signaling Technology (Danvers, MA, USA), were used: phospho-Smad2 Ser^245/250/255^), Smad2, and α-tubulin as well as the following from Santa Cruz Biotechnology (Dallas, TX, USA): Snail1 and Snail2. Appropriate secondary horseradish peroxidase-labeled antibodies (Jackson ImmunoResearch, West Grove, PA, USA) were applied at 40 ng/ml in blocking agent for 1 h at room temperature. Blots were developed with ECL Advance Detection Reagents (GE Healthcare, Boston, MA, USA) and imaged with a Chemi-Smart gel-doc system (Vilber Lourmat, Marne-la-Vallée, France). Band quantification was performed with Labworks Software (UVP, Upland, CA, USA) and protein expression data analyzed using Prism 5 (GraphPad, La Jolla, CA, USA).

### EMT induction

EMT was induced by adding StemXVivo EMT Inducing Media Supplement (R&D Systems, Minneapolis, MN, USA) and monitored per manufacturer’s recommendations [41]. Briefly, cells were gently detached from culture dishes using a dissociation solution, centrifuged, and re-suspended in warmed culture media containing StemXVivo EMT Inducing Media Supplement. Cell cultures were incubated at 37 °C and 5% CO_2_, and media changed every two days. EMT induction was completed five days after plating.

### Immunocytochemistry

Protein expression analysis by immunocytochemistry was performed using the Human EMT 3-Color Immunocytochemistry kit (R&D Systems Inc., Minneapolis MN, USA). Briefly, cells were fixed in 4% paraformaldehyde, incubated in a blocking buffer made up of 1% bovine serum albumin (BSA), 10% rabbit serum, and 0.3% Triton X-100 in 1X PBS. Confluent areas on slides were marked using PAP pens. Samples were further incubated in blocking buffer containing conjugated antibodies to human Snail-NL557, E-Cadherin-NL637, and Vimentin-NL493, diluted according to the manufacturer’s instructions. Nuclei were counterstained with Hoechst dye (Life Technologies Inc., Burlington ON, Canada) and mounted with Prolong Gold Antifade Reagent (Life Technologies Inc., Burlington ON, Canada). Slides were allowed to dry in the dark overnight, and the samples were imaged using an Olympus Fluoview FV1000 confocal laser scanning microscope (Olympus Inc., Waltham, MA, USA). A minimum of three independent experiments were performed for each condition. Normalized fluorescence intensity was calculated using ImageJ (v1.48, U.S. National Institute of Health) as previously described [42]. In summary, ImageJ software was used to draw an outline around each cell and fluorescence was quantified. Normalized fluorescence intensity was calculated by dividing the total fluorescence in each replicate by the number of cells. The mean and standard error of normalized fluorescence intensity from a minimum of three replicates was calculated.

### Microarrays

GeneChip PrimeView Human Gene Expression Microarrays (Affymetrix, Santa Clara CA, USA) were used to quantify changes in gene expression. Signal intensity files were pre-processed using robust multichip average normalization [43]. Microarray data was analyzed using Biometric Research Branch—ArrayTools Version 4.5.0 (National Cancer Institute, USA) [44] and Partek Genomics Suite Version 6.6 (Partek Incorporated, Missouri, USA). For each cell line, microarrays were run for 3 independent replicates of static and flow-exposed cells and differentially expressed genes (fold change ≥ 1.5 and *p*-value ≤ 0.01) were identified by analyzing class comparisons between static and flow samples. Similarly, gene set enrichment analyses were performed against gene sets from the Molecular Signature Database (Broad Institute, USA), with at least 5 differentially expressed genes in a particular Gene Ontology category at a *p*-value ≤ 0.05 considered statistically significant [45]. All microarray experiments were performed at the Arnie Charbonneau Cancer Institute Microarray Facility (Alberta Health Region, Canada). Raw microarray multimedia fusion array and Affymetrix probe result files for each replicate are provided as Additional files 1–49.

### Interaction network analysis

The Cytoscape (version 3.0) software platform was used to visualize gene interaction networks [46, 47]. Differentially expressed genes between static and flow-exposed breast cancer cells were mapped onto a human interactome network obtained from integrated complex traits networks (iCTNet), Version 1.0 [47]. The BiNGO and MCODE plugins were used to assess enrichment of nodes for biological processes recorded in the Gene Ontology database [48, 49].

### Smad2 and Smad3 siRNA transfections

200,000 MDA-MB-231 cells were seeded in T-75 culture flasks for 24 h before being transfected with 25 nM Smad2 or Smad3 siRNA (Santa Cruz Biotechnology) for 4 h in 0.3% PepMute siRNA Transfection Reagent (SignaGen Laboratories, Rockville, MD, USA). Afterwards, a media change was made, and transfected cells were re-suspended and plated on glass slides pre-coated with 145 µg/mL Rat Tail collagen I (Life Technologies Inc., ON, Canada). Cells were left to grow for 24 h before being used in cell culture, flow, and PCR experiments as previously described. The methodologies for optimizing siRNA transfections, control and transfection efficiency experiments are described in Supplemental Methods.

### Clinical patient data analysis

Level 3 mRNA expression data from The Cancer Genome Atlas (TCGA) was obtained from Synapse (http://synapse.org; syn1461151) [50]. Gene expression data was measured using the Illumina HiSeq 2000 RNA Sequencing version 2 analysis platform (RNA-Seq by Expectation Maximization, RSEM). This dataset contains whole-genome expression data for 104 healthy volunteers, 317 luminal A, 93 luminal B, 26 HER2, and 81 basal breast cancer patients. Breast cancer subtype classification was based on immunohistochemical expression of estrogen receptor (ER), progesterone receptor (PR) and human epidermal receptor 2 (HER2) [36, 51]. Gene expression was compared between subtypes using box plots showing the median and interquartile ranges. Prognostic value of genes differentially expressed upon flow stimulation was evaluated using Kaplan–Meier curves (http://kmplot.com/analysis/) to compare relapse-free survival times in a data set containing 2878 breast cancer patients [52]. Statistical significance was determined by the log-rank test.

### Adhesion assay

A Human Umbilical Vein Endothelial Cell (HUVEC) monolayer was obtained by seeding 250,000 cells per well in gelatin-coated 6-well culture dishes, grown for two days and activated with 150 ng/mL of TNF-α for 5 h. HUVECs were obtained from Lonza (Walkersville MD, USA) and cultured per recommendations. MDA-MB-231 and MCF-7 cells were cultured, and flow preconditioned as described above. Flow-stimulated and unstimulated cells were non-enzymatically detached from glass plates, re-suspended in serum free media, and added to the HUVEC monolayers at a density of 200,000 cells per well. Cancer cells were left to adhere on the endothelial monolayer for 90 min, after which non-adherent cells were carefully washed twice with HBSS and cells were fixed with 4% PFA for 10 min. Adherent breast cancer cells from four independent wells (five fields per well) were counted and used to quantify relative adhesion as the ratio of adherent cancer cells per endothelial cells present in a field.

### Adhesion experiments under flow

A HUVEC monolayer was cultured on glass slides by seeding 200,000 cells per slide and grown for two days. Slides were pre-coated with 145 µg/mL Rat Tail collagen I (Life Technologies Inc., ON, Canada). Following activation with 150 ng/mL of TNF-α for 5 h, slides were embedded in a parallel-plate flow chamber as previously described. Flow-stimulated and unstimulated cells (200,000 cells/ml) were suspended in serum free media, loaded onto a syringe, and mounted on an infusion pump (Harvard Bioscience, Holliston, MA, USA) connected to the endothelial monolayer. Cancer cells were pumped over the endothelial monolayer at a shear rate of 16 s^−1^ for 10 min. Each experiment was replicated four times. Pictures were taken at several time points and several locations on the slides and used to quantify relative adhesion as the ratio of adherent cancer cells per endothelial cells present in a field.

### Cell migration and invasion assays

For migration assays, flow-stimulated or unstimulated MDA-MB-231 cells were plated in 6-well Polycarbonate Membrane Transwell® Inserts with 8 µm pores (Corning Inc., Lowell, MA, USA) as previously described [53, 54]. Briefly, 300,000 cells were resuspended in 1.5 mL DMEM media containing 0.5% FBS and seeded to the upper compartment of the transwell. To the lower compartment, 2.6 mL of DMEM containing 0.5% FBS and 40 µg/ml Rat Tail Collagen I (Life Technologies Inc., ON, Canada) was added, and incubated for 2.5 h. After this, inserts were carefully taken out and with the use of cotton swabs, cells that had not migrated through the pores and remained on the upper side of the filter membrane were gently removed. Migrated cells were imaged using an Olympus FV1000 confocal microscope (Olympus Inc., Waltham, MA, USA) with a 10 × objective. Cells that migrated through the membrane were counted in at least three random microscopic fields. Three separate experiments were performed for each condition and statistical significance was determined using the Student *t-*test. For invasion assays, the same experimental procedure as for transwell migration assays was used except that the 6-well inserts were coated with Growth Factor-Reduced BD Matrigel Matrix (BD Biosciences, San Jose, CA, USA) for 1.5 h prior to being seeded with cells.

## Results

### Fluid flow affects breast cancer cell line gene expression and enriches cellular processes involved in metastasis

**Fig. 1:**
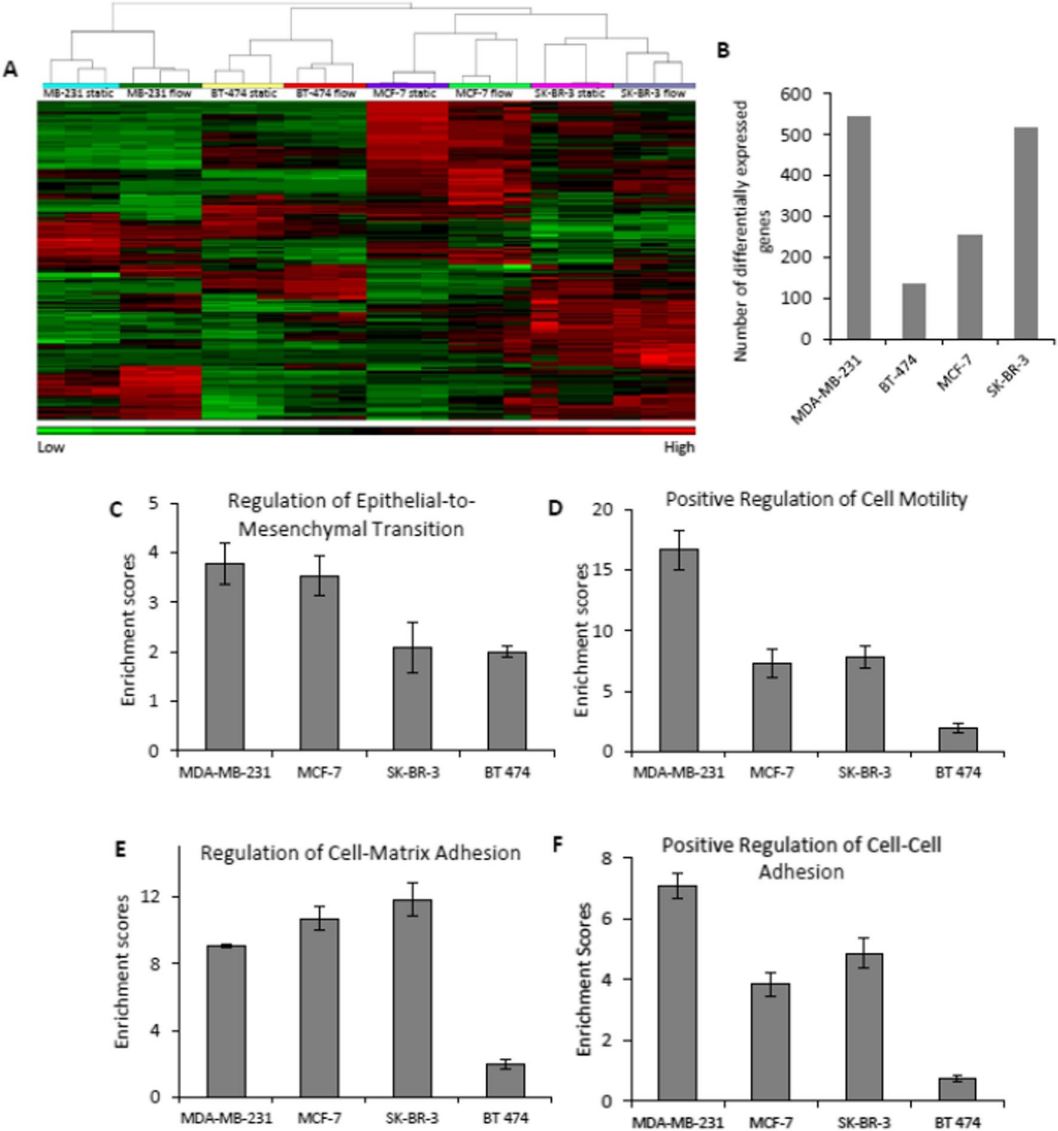
**a** Heat map of differentially expressed genes between static and flow-exposed MDA-MB-231, BT-474, SK-BR-3 and MCF-7 breast cancer cells; **b** Number of differentially expressed genes between static and flow-exposed breast cancer cells (fold change ≥ 1.5 and *p* ≤ 0.01); Exposure of breast cancer cells to fluid flow enriched molecular processes involved in metastatic progression, including Regulation of Epithelial-to-Mesenchymal Transition (**c**), Positive regulation of cell motility (**d**), Positive regulation of cell–cell adhesion (**e**), and Regulation of cell–matrix adhesion (**f**). GO categories with enrichment scores ≥ 2 and *p* ≤ 0.05 were considered statistically significant. For each cell type, microarrays were run for 3 biological replicates of static and flow-exposed cells

To investigate the effect of fluid flow on breast cancer cell line gene expression, BT-474, MCF-7, MDA-MB-231, and SK-BR-3 breast cancer cells were exposed to fluid flow at 1 Pa derived from the parallel-plate bioreactor system. Significant differences in gene expression profiles between flow-exposed and non-exposed cells were observed upon analysis of whole-genome expression data using microarrays (Fig. 1a). At least 150 genes were differentially expressed in each breast cancer cell line upon flow exposure (Fig. 1b). The MDA-MB-231 basal cell line had the most differentially expressed genes upon flow stimulation (Fig. 1b). Basal breast cancer is one of the most aggressive subtypes of breast cancer, with unique biology characterized by an early pattern for metastasis [51, 55].

**Fig. 2:**
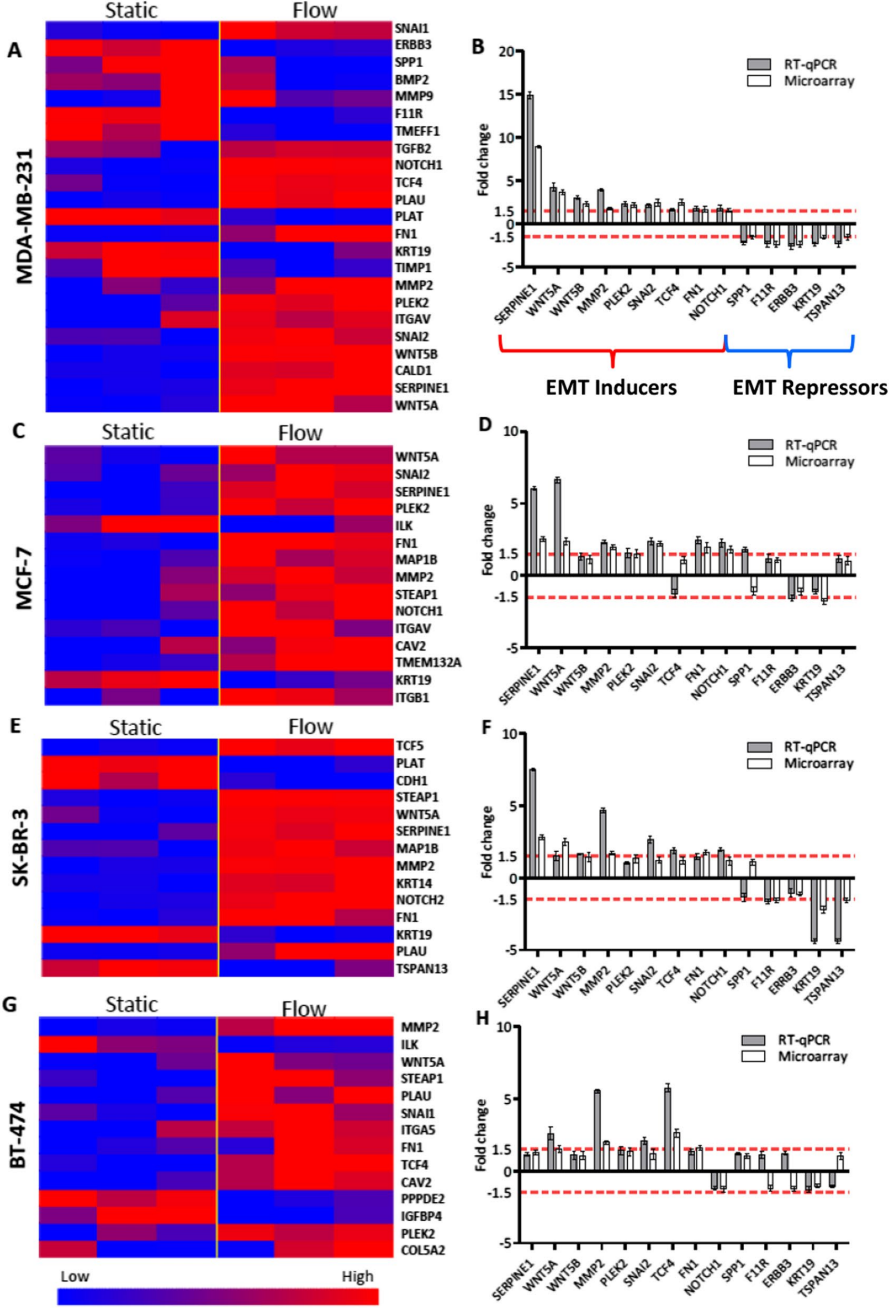
EMT-inducers were consistently upregulated, and EMT-repressors were downregulated after exposing breast cancer cells to fluid flow, as shown by heat maps of static and flow-exposed breast cancer cells (**a, c, e, g**). Microarray expression data for each cell line was validated with qPCR (**b, d, f, h**). Three independent replicates were run for each condition and relative gene expression was calculated using the comparative cycle threshold method. Red dotted lines indicate limits of fold change for differentially expressed genes

To identify key molecular functions and biological processes that were significantly affected by flow exposure, we performed gene set enrichment analysis using microarray expression data for each cell line [45]. Gene subsets involved in cellular processes that contribute to metastatic progression of carcinomas were profoundly enriched in most cell lines upon flow stimulation. In all breast cancer cells that were exposed to flow, we observed significant enrichment of genes involved in regulation of EMT (Fig. 1c), positive regulation of cell motility (Fig. 1d), regulation of cell–matrix adhesion (Fig. 1e), and positive regulation of cell–cell adhesion (Fig. 1f). In addition, we observed upregulation of several collagens, integrins and other cell adhesion molecules, including FN1 and ALCAM, suggesting modulation of adhesive properties in cancer cells upon flow exposure (Figs. 2 and 6d, e).

### Fluid flow upregulates expression of EMT genes and proteins in breast cancer cells

To further examine EMT regulation upon flow stimulation, we analyzed expression of genes in an EMT gene set created from the PCR Array for Human Epithelial-to-Mesenchymal Transition. Microarray data analysis revealed heat maps of differentially expressed EMT genes upon flow stimulation for each breast cancer cell line, including consistent upregulation of EMT inducers such as FN1, SNAI2, SERPINE1, NOTCH1, TCF4, MMP2, PLAU, WNT5A and WNT5B in most flow stimulated cells (Fig. 2a, c, e and g). EMT repressors, such as KRT19 and TSPAN13 were downregulated in several cell lines. Consistent upregulation of EMT inducers and downregulation of EMT repressors was confirmed by qPCR and shown in Fig. 2b, d, f and h. Further, we observed that the flow-induced upregulation of SNAI2 was sustained through 24 h after flow was stopped in MDA-MB-231 cells (Additional file 49: Fig. S1A) and at lower shear stresses of 0.2 Pa and 0.6 Pa (Additional file 49: Fig. S1B).

**Fig. 3:**
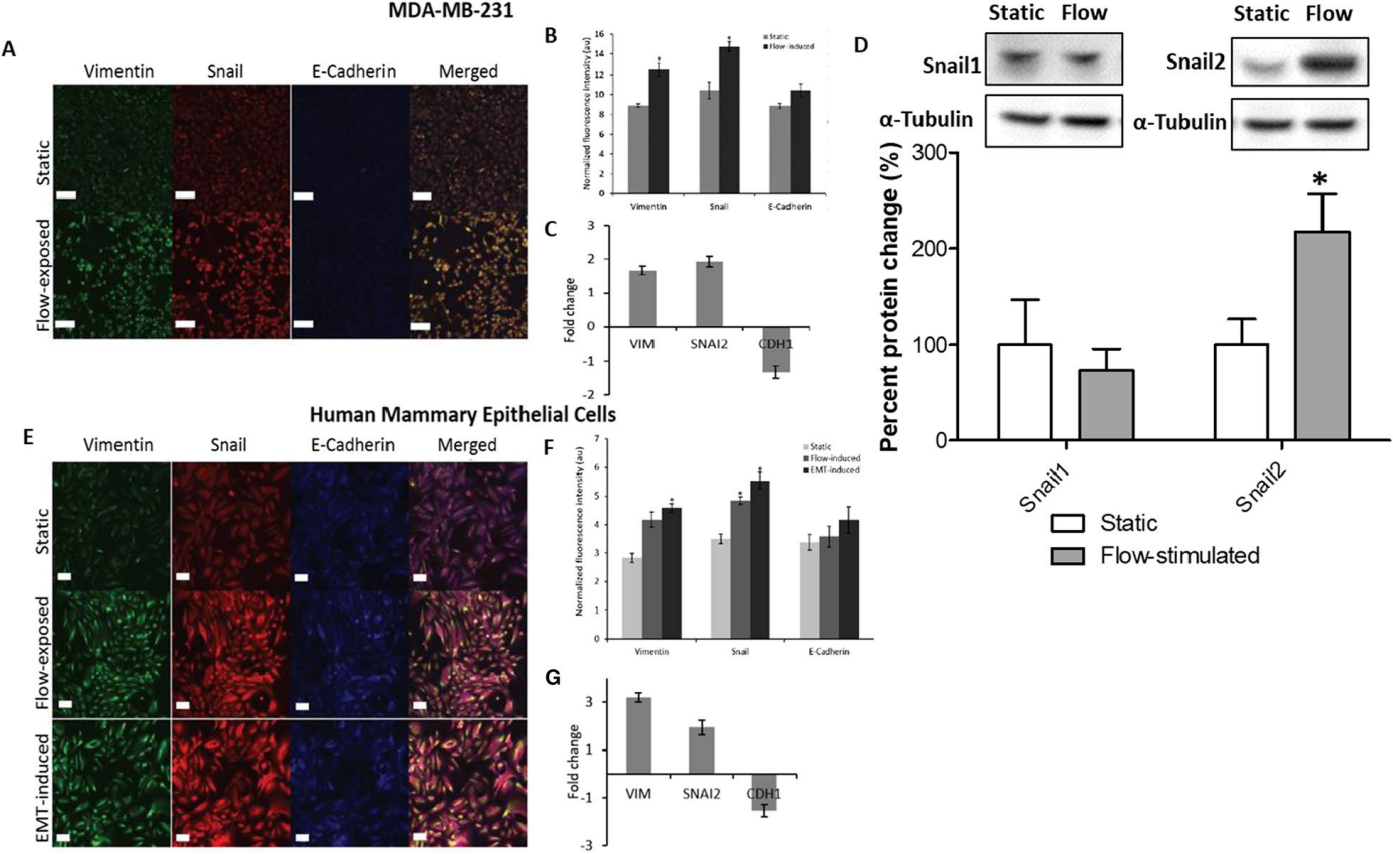
Representative confocal microscopy images of static (*n* = 8) and 20 h flow-exposed (*n* = 8) MDA-MB-231 cells (**a**), scale bars = 90 µm; and static (*n* = 8), 20 h flow-exposed (*n* = 8) and EMT-induced (*n* = 6) Human Mammary Epithelial Cells (HMECs) (**e**), analyzed for EMT by simultaneously staining with fluorochrome-conjugated antibodies for Vimentin (green), Snail (red) and E-Cadherin (blue). Scale bars = 60 µm. Normalized fluorescence intensity of static and flow-exposed MDA-MB-231 cells (**b**)**,** and HMECs (**f**). Fluorescence intensity was normalized to number of cells in each microscopic field and compared to intensity in static controls. Data shown represent the means ± standard errors of the means of data from at least 3 samples for each condition. au = arbitrary units. Quantitative RT-PCR analysis of VIM, SNAI2 and CDH1 expression between static and flow-exposed MDA-MB-231 cells (**c**) and HMECs (**g**). Triplicate wells were run for every sample and B2M was used as the reference gene. Representative immunoblots showing no differential expression of Snail1 and overexpression of Snail2 following exposure to fluid flow in MDA-MB-231 cells (**d**). α-tubulin was used as the protein loading control. Lower panels show densitometric analysis of the blots normalized to α-tubulin. Data shown represent the means ± standard error of the means of data from at least 3 samples for each condition. (*) *p* < 0.05

Further, we wanted to compare static and flow-derived EMT protein expression profiles for MDA-MB-231 cells using a Human EMT 3-Color Immunocytochemistry Kit. Promotion of EMT in flow stimulated MDA-MB-231 cells was revealed by concurrent upregulation of Snail and Vimentin (Fig. 3a and b). These findings were consistent with qPCR expression data for VIM and SNAI2 (Fig. 3c), the genes that respectively code for Vimentin and Snail. Western blot analyses supported these findings by revealing upregulation of snail2 and no differential expression of snail1 (Fig. 3d). E-cadherin was expressed at low levels in MDA-MB-231 cells (Fig. 3a). We also observed slight or no downregulation of CDH1 and upregulation of CDH2 upon flow exposure of MCF-7 and BT-474 cells (data not shown), suggesting progression to a more mesenchymal phenotype.

Next, we compared the expression levels of E-cadherin, Vimentin and Snail in HMECs that were chemically induced for EMT, exposed to fluid flow, or cultured in static conditions. Snail and Vimentin were upregulated in both flow-exposed and EMT-induced cells compared to static controls, while E-cadherin showed no significant differences in expression between all three conditions (Fig. 3d and e). Quantitative PCR data revealed upregulation of SNAI2 and VIM, and insignificant changes in CDH1 in flow-exposed HMECs compared to static controls (Fig. 3f). These results suggest that exposing HMECs to fluid flow stimulates progression towards an EMT phenotype similarly to chemical induction.

### Smad3 and not smad2 knockdown affects flow-induced upregulation of SNAI2

**Fig. 4:**
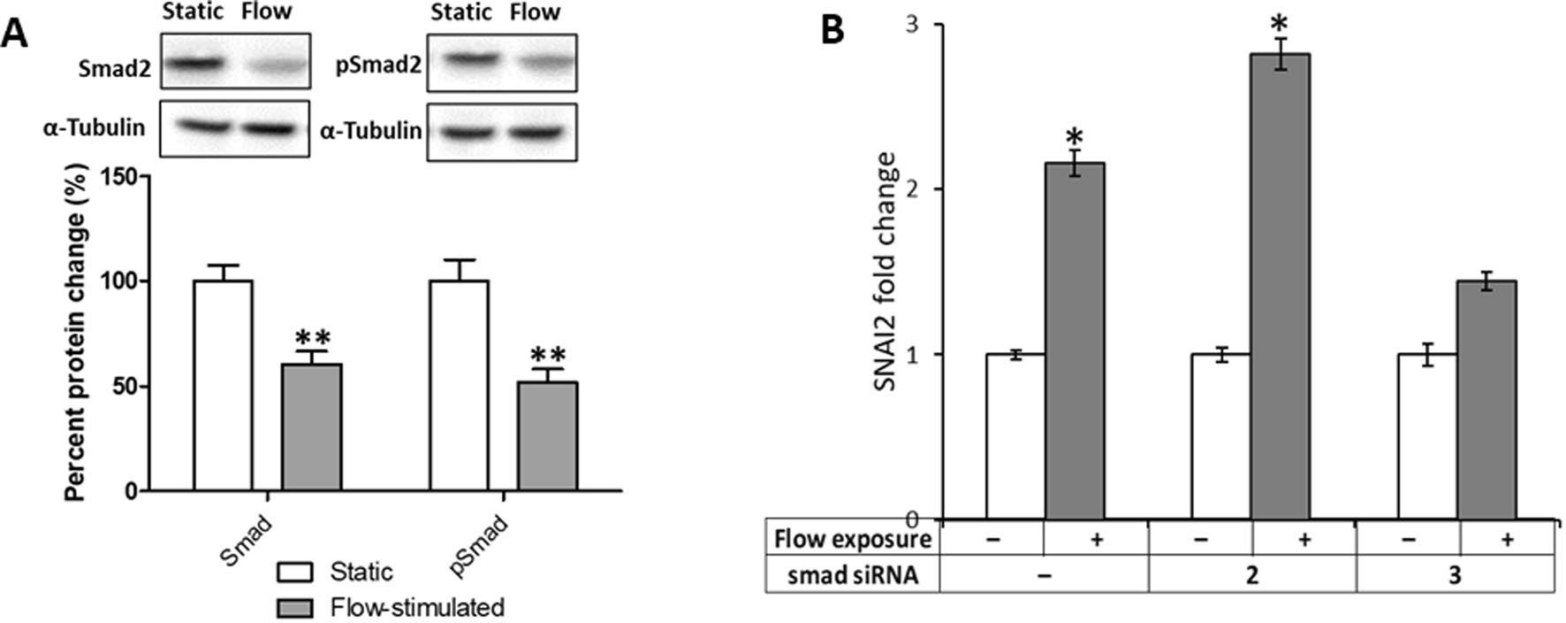
**a** Smad2 protein expression (total SMAD2 and pSmad2-linker) as determined by western blotting in MDA-MB-231 cells. **b** Quantitative RT-PCR analysis of SNAI2 expression in MDA-MB-231 cells transfected with control (–), Smad2 or Smad3 siRNA and stimulated with fluid flow for 20 h. Control siRNA (negative control) consisted of a scrambled sequence. Fold changes were calculated using the comparative cycle threshold method and normalized to expression in unstimulated controls. Statistical significance was determined using the Student’s *t*-test and the threshold was set at *p* < 0.05. Duplicate wells were run for every sample and beta-2-microglobulin was used as the reference gene. Results in the graphs are expressed as mean ± standard error of the means (*, *p* < 0.05) from at least three samples for each condition

Smad2/3 is a known modulator of EMT in cancer and is also known to be flow responsive. Smad2 levels (total and linker phosphorylated) were measured in MDA-MB-231 cells and found to decrease upon flow exposure (Fig. 4). To investigate involvement of Smad2 or Smad3 in flow-induced upregulation of SNAI2, MDA-MB-231 cells were transfected with Smad2 or Smad3 siRNA and used to quantify relative expression of flow-stimulated cells with respect to expression in unstimulated controls. SNAI2 was upregulated 2.2 and 2.8 folds upon flow stimulation in cells transfected with control and Smad2 siRNA, respectively. Insignificant differential expression (1.4 folds) was observed between the two conditions when cells were transfected with Smad3 siRNA. As shown in Additional file 49: Table S1, transfection with Smad2 and Smad3 resulted in knockdown of 91% and 86%, respectively. Results of siRNA optimization and control experiments are presented in Additional file 49: Figs. S2 and S3.

### Network analysis revealed key genes involved in cell flow responses

**Fig. 5:**
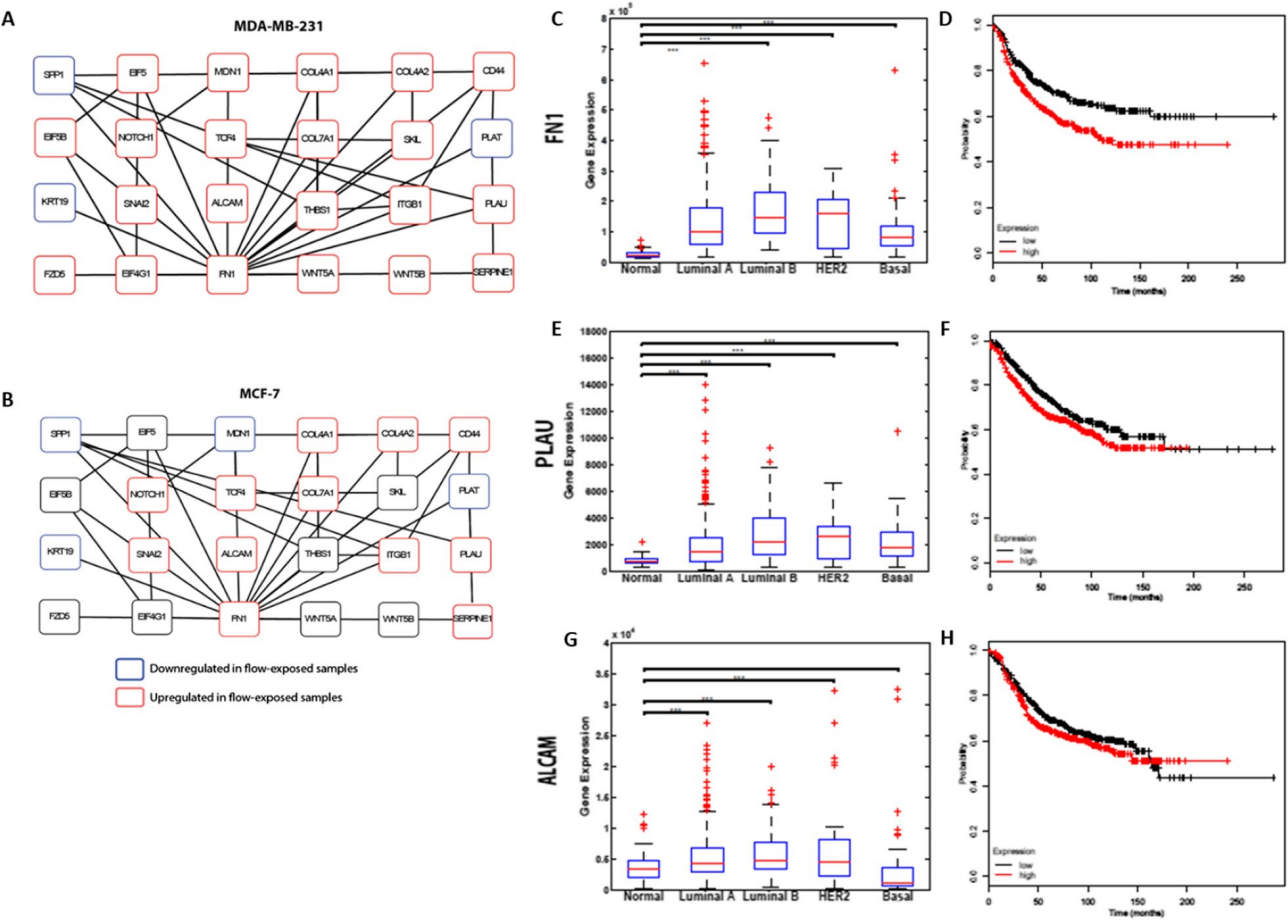
Sub-network of Fibronectin (FN1) and its differentially expressed first degree neighbours showing expression between static and flow-exposed MDA-MB-231 (**a**) and MCF-7 (**b**) breast cancer cells. Red and blue nodes respectively represent genes that were upregulated and downregulated upon flow exposure. Box plots showing expression of FN1 (**c**), PLAU (**e**), and ALCAM (**g**) between normal volunteers and patients with several stratifications of breast cancer. Expression data was obtained from TCGA (Synapse ID 1461151) and consisted of 104 healthy volunteers (normal), 317 luminal A, 93 luminal B, 26 HER2 and 81 basal patients. The boxes show the median and the interquartile range. The whiskers show the minimum and maximum. *** = *p* value ≤ 0.001. Kaplan–Meier curves of relapse-free survival times of breast cancer patients with lymph node positive status (*n* = 936), stratified by FN1 (**d**), PLAU (**f**) and ALCAM (**h**) expression. Data was obtained from http//kmplot.com/analysis and statistical significance was determined by the log-rank test. Survival plots for FN1 and PLAU are statistically significant (*p* ≤ 0.05) while that of ALCAM is not statistically significant (*p* = 0.18)

To visualize molecular interaction networks that are possibly involved in promoting EMT and cell adhesion in flow stimulated breast cancer cells, we imported and analyzed gene expression data for flow-exposed and static controls using the Cytoscape software platform. A highly curated human interactome network was downloaded using iCTNet (version 1.0) [47]. The network corresponding to the differentially expressed genes (with fold changes ≥ 5) and their first degree neighbours between static and flow-exposed MDA-MB-231 cells was highly clustered, indicating a high degree of modularity of protein networks when breast cancer cells are exposed to flow. Due to overexpression of FN1 in most flow-exposed cells and its roles in EMT and cell adhesion, we analyzed its interaction sub-network to better understand how flow exposure potentially affects metastatic events in breast cancer cells. The FN1 sub-networks for MDA-MB-231 (Fig. 5a) and MCF-7 (Fig. 5b) revealed functional links through which overexpression of FN1 possibly promotes EMT and adhesion in breast cancer cells. This was observed through interactions between FN1 and its differentially expressed first degree neighbours, including EMT genes such as SNAI2, NOTCH1, TCF4, PLAU, SPP1 and KRT19 as well as cell adhesion genes such as ALCAM, COL4A1, COL4A2, COL7A1 and ITGB1.

### Bioreactor-derived expression changes recapitulate clinical breast cancer gene expression profiles

To investigate the relevance of our findings to progression of human breast cancer, we analyzed gene expression data of FN1 and two of its first-degree neighbours (PLAU and ALCAM), using data from The Cancer Genome Atlas (TCGA) [50]. We found that FN1, PLAU and ALCAM were upregulated in patients with most subtypes of breast cancer compared to expression in healthy volunteers (Fig. 5c, e and g). We also observed upregulation of FN1, PLAU and ALCAM in patients at most stages of breast cancer compared to expression in healthy individuals.

Next, the prognostic value of FN1, PLAU and ALCAM was evaluated in a microarray data set of breast tumors from 2878 patients [52]. Kaplan–Meier curves of relapse-free survival times of breast cancer patients with lymph node positive status, revealed patients with high FN1 and PLAU expression (the median value in the entire patient dataset was used as the cut off for low and high expression) have reduced survival profiles compared to patients with low FN1 and PLAU expression (Fig. 5d and f). Kaplan–Meier survival plots for ALCAM expression showed a similar pattern (Fig. 5h), although not statistically significant (*p* = 0.18).

### Fluid flow stimulation increases adhesive, migratory and invasive abilities of breast cancer cells

**Fig. 6:**
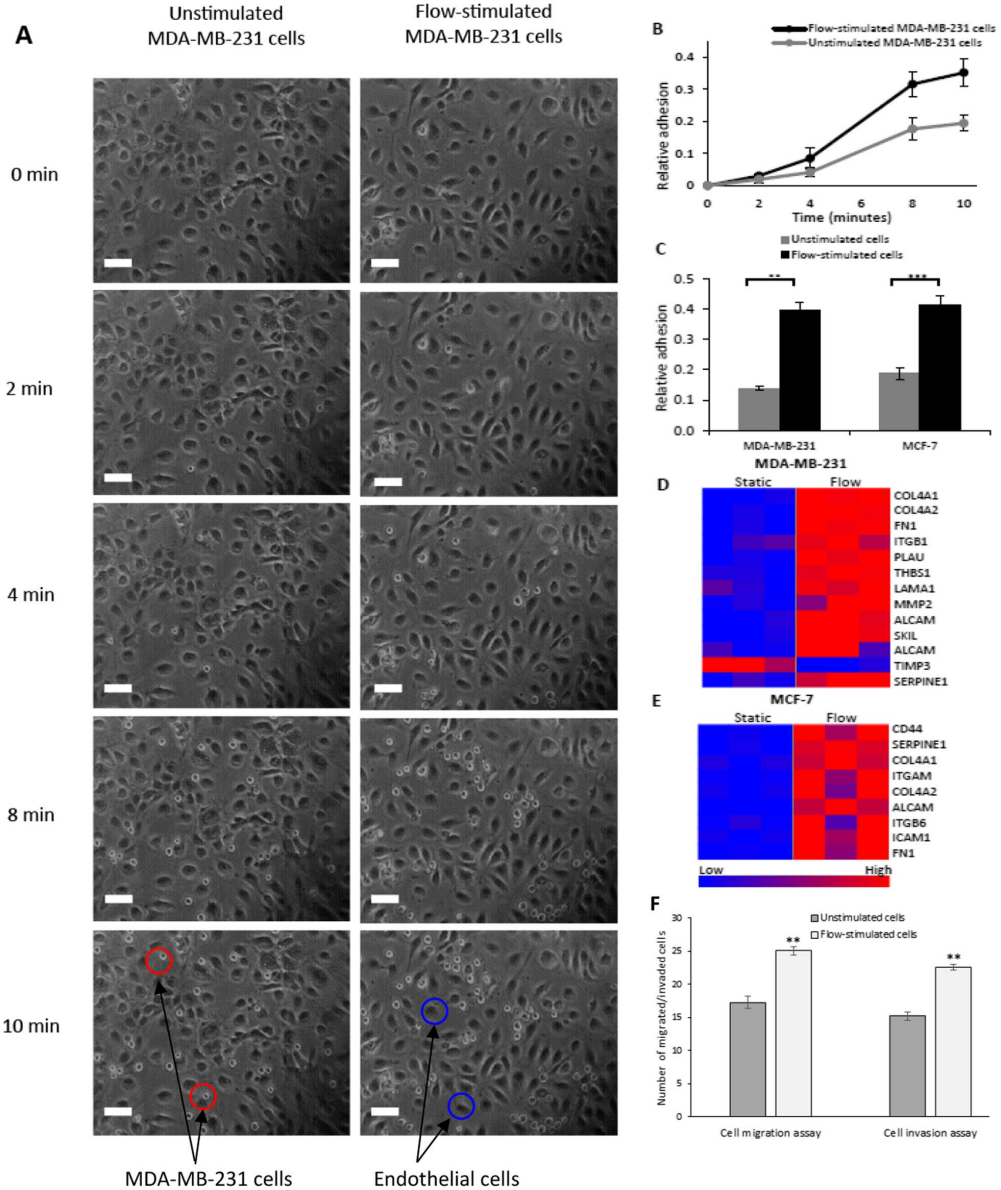
**a** Representative phase contrast images of unstimulated and flow-stimulated MDA-MB-231 cells adhering to an endothelial monolayer at several time points. MDA-MB-231 cells present with light grey nuclei while endothelial cells present with dark grey nuclei. The phase contrast images are representative of 4 independent experiments with similar results. Scale bars = 80 µm. Relative adhesion of flow-stimulated (black line) and unstimulated (grey line) cells in flow (**b**) and static (**c**) adhesion studies. Relative adhesion is defined as average number of adhered cancer cells per endothelial cells in a field. Results in the graphs are expressed as mean ± SE (**, *P* < 0.01; ***, *P* < 0.001). Heat maps of differentially expressed cell adhesion genes (fold change ≥ 2 and *p* ≤ 0.01) between static and flow-exposed MDA-MB-231 (**d**) and MCF-7 (**e**) breast cancer cells. **f** Comparison of migration and invasion of flow-stimulated and unstimulated MDA-MB-231 breast cancer cells in transwell assays. Cells in 3 random microscopic fields were counted for each group. The results presented are an average of three random microscopic fields from three independent experiments

We then evaluated the ability of flow-stimulated and unstimulated MDA-MB-231 and MCF-7 cells to adhere to an endothelial monolayer. Figure 6a shows representative cancer cell—endothelial cell adhesion images obtained after unstimulated and flow-stimulated MDA-MB-231 cells were flowed over an endothelial monolayer. Flow-stimulated MDA-MB-231 cells adhered more to endothelial cells than unstimulated cells at all time points beyond 4 min (Fig. 6a and b). In addition, flow-stimulated MDA-MB-231 and MCF-7 cells showed greater adhesion to endothelial monolayers in static adhesion experiments compared to unstimulated cells (Fig. 6c). We also observed significant upregulation of a key panel of cancer cell adhesion genes upon flow stimulation of MDA-MB-231 (Fig. 6d) and MCF-7 (Fig. 6e) cells. Transwell migration and invasion assays were used to investigate migratory and invasive abilities of flow-stimulated and unstimulated cells. Figure 6f shows that exposure of MDA-MB-231 cells to fluid flow increases their abilities to migrate and invade through transwell membranes. Taken together, these results reveal a potential role of fluid flow exposure in potentiating breast cancer cell migration, adhesion to endothelial cells and invasion through membranes at metastatic sites.

## Discussion

Our results show that exposure to fluid forces on the order of 1 Pa promotes EMT and adhesion of breast cancer cells to endothelial cells. This supports incorporation of the effect of fluid flow in studies seeking to understand development of this complex disease, whose progression is characterized by mechanical interactions between tumor cells and the various microenvironments they encounter [7]. Imperative to metastasis is tumor cell detachment from the primary tumor, during which the process of EMT contributes to increased motility and adhesiveness to endothelial cells lining blood vessels of metastatic sites [17, 18]. Before invading secondary organs, tumor cells must encounter fluid shear stresses produced by the slow interstitial flow in the tumor microenvironment believed to be on the order of 0.01 Pa, and the hemodynamic shear forces in the blood stream which range from 0.05 to 3 Pa [6, 8]. In this study, we used a bioreactor system consisting of a parallel-plate flow chamber to expose various stratifications of breast cancer cells to moderate fluid shear stress (1 Pa), modelling exposure of cells to the flow conditions created in the vascular microenvironment. Our findings reveal an effect of fluid flow on breast cancer cell line gene expression by promotion of EMT and adhesion to endothelial cells.

We also observed consistent upregulation of EMT inducers, including SNAI2, NOTCH1, FN1, VIM, PLAU, MMP2, WNT5A and TCF4, and downregulation of EMT repressors such as TSPAN13 and KRT19 (Fig. 2) upon flow stimulation. Compared to expression in static culture, SNAI2 remained upregulated up to 24 h after flow was stopped in MDA-MB-231 cells and at lower shear stresses (0.2 Pa and 0.6 Pa). Flow stimulation also resulted in EMT induction in mammary epithelial cells similarly to chemical induction (Fig. 3). These results suggest a potential effect of fluid flow on the metastatic ability of breast cancer cells by promotion of EMT, and support results of previous studies on shear stress affecting metastatic events in other types of cancer [10, 11, 14, 24, 56].

Few studies have examined how fluid forces in the physiological range of those experienced in the vascular microenvironment affect cellular events during breast cancer progression [24, 57]. Here we show that exposure to fluid flow on the order of 1 Pa promotes EMT in all molecularly classified subtypes of breast cancer. Further, we show that this effect is more pronounced in the basal MDA-MB-231 cells, representing the most clinically aggressive subtype of breast cancer. This subtype is highly studied due to its unique biology, overall poor prognosis, early pattern for metastases and relative lack of therapeutic targets compared to the other subtypes [51, 58–60]. Previous in vitro studies in which cancer cells were exposed to fluid shear stresses in the range of those encountered in the tumor microenvironment have demonstrated promotion or induction of EMT [12, 24, 61–63]. These lower shear stresses promoted internalization of E-cadherin in metastatic esophageal cancer cells [61, 64], highlight the importance of Caveolin-1 as a key mechanosensor during hematogenous metastasis [62], and induced EMT in 3D ovarian cancer nodules by downregulating E-cadherin and upregulating Vimentin both at the transcript and protein levels [14]. Tchafa et al. [10] reported an effect on EMT in breast cancer cells following stimulation by fluid flow in the magnitude of those experienced in the tumor microenvironment. In that study, an alteration in the mechanism of interstitial flow-induced invasion in HER2-expressing breast cancer cells following EMT induction was reported [10]. However, the HER2-enriched subtype of breast cancer is just one of the four major subtypes of breast carcinomas [36, 50].

Network analysis revealed key genes involved in flow responses including FN1, PLAU and ALCAM. FN1 is an extracellular matrix (ECM) glycoprotein that binds to membrane-spanning receptor proteins and plays essential roles in EMT [65], cell adhesion [66], proliferation [65], and migration [66, 67]. PLAU encodes an enzyme that is involved in ECM proteolysis, tumor cell migration and proliferation [56, 68]. Its receptor, PLAUR regulates integrin function by mediating cell signaling in response to PLAU binding [69]. Several studies have shown that the FN1-binding integrin (α5β1) co-immunoprecipitates with PLAUR, suggesting FN1 binding to its receptors potentially enhances PLAU-PLAUR signaling during EMT promotion [27, 69, 70]. In addition, increased secretion of PLAU results in activation of MMPs, which are important in initiating or promoting EMT [71, 72].

Flow stimulation also resulted in differential expression of cell adhesion genes, including FN1 and ALCAM. Besides being upregulated during EMT, FN1 plays important roles during subsequent adhesion of cancer cells to endothelial cells [66]. Binding of FN1 to integrin α5β1 triggers integrin-mediated intracellular signals culminating in increased adhesion to endothelial cells [34, 66, 73]. Interestingly, our network analysis revealed an interaction between FN1 and ALCAM. As a type 1 transmembrane glycoprotein of the immunoglobulin superfamily, ALCAM functions as a cell surface sensor and promoter of interactions between cancer and endothelial cells [32]. Upregulation of ALCAM in flow-stimulated breast cancer cells suggested potential roles of blood flow in promoting cancer cell adhesion to endothelial cells. Finally, our hypothesis was supported by significant adhesion of flow-stimulated breast cancer cells to TNF-α stimulated endothelial cells in static and laminar flow adhesion assays.

When evaluated in a clinical cohort, we observed significant increase in expression of FN1, PLAU and ALCAM in patients with most subtypes of breast cancer compared to healthy volunteers. Kaplan–Meier curves of relapse-free survival times of breast cancer patients with lymph node positive status, stratified by FN1 (Fig. 5b), and PLAU (Fig. 5d) expression levels, demonstrate that expression changes obtained from our bioreactor system recapitulate clinical breast cancer gene expression profiles. We agree with the potential applications of FN1, PLAU and ALCAM as prognostic and/or predictive biomarkers for breast cancer based on their differential expression upon flow stimulation, interactions with other genes involved in EMT and cell adhesion, relapse free survival analyses and overexpression in most subtypes and at most stages of breast cancer when compared to expression in healthy volunteers. The clinical ramifications of these findings have been partly demonstrated through the use of EMT gene expression profiles as useful prognostic markers for disease-free survival in patients with hepatocellular carcinoma [74]. PLAU is a mesenchymal marker that is upregulated after an EMT and its potential application as a biomarker for breast cancer has been evaluated and presented by Schmitt et al. [75]. ALCAM has also been evaluated as a prognostic biomarker in a variety of cancers due to its overexpression in patients with breast [32, 34], ovarian [33], pancreatic [76], and non-small-cell lung cancers [77].

Multiple signaling pathways cooperate in the initiation and progression of cancer, notably TGF-β, WNT-β-catenin, Notch and RAS-MAPK pathways. We observed differential expression of genes in these pathways and network analysis revealed key interactions that possibly contribute in EMT promotion and increased adhesiveness to endothelial cells during metastatic progression. These findings support previous studies that reported modulation of cell signalling pathways involved in tumor metastasis in response to fluid shear stress exposure in other cell types [24, 59, 62, 68, 69]. We found TGF-β/Smad signaling to be of particular interest as this pathway has been found to be a major inducer of EMT during embryonic development and cancer progression [23, 78, 79]. Interestingly, several TGF-β/Smad signaling and EMT genes, including SNAI2, were significantly upregulated in most breast cancer cells exposed to fluid forces, similar to what has already been reported in TGF-β-driven EMT in several cell lines [80, 81]. SNAI2 plays an important role in EMT and cell adhesion in breast cancer, and its upregulation is associated with breast cancer aggressiveness [82]. In siRNA knockdown studies, we observed that Smad3 knockdown successfully abrogated flow-induced overexpression of SNAI2 while Smad2 knockdown did not. These results suggest an important role of Smad3 in transducing the mechanical forces from fluid flow to alter expression patterns of SNAI2 in breast cancer cells and support previous work by Thault et al. (2009) in which smad3, but not Smad2 knockout effectively blocked TGF-β-induced EMT response through SNAI2 signaling [80, 83]. This stance is further supported by the observation that TGF-β-driven EMT in hepatocytes is dependent on Smad3, and not Smad2 signaling [84].

## Conclusions

Using the parallel-pate flow chamber system, we created an experimental model of breast cancer progression and identified biomarkers that were differentially expressed in patient populations. Understanding how biophysical forces such as shear stress affect cellular processes involved in metastatic progression of breast cancer is important for identifying new molecular markers for disease progression, and for predicting metastatic risk. These findings support using flow-based models to further investigate how fluid environments affect cancer cells and their metastatic abilities. Taken together, our results demonstrate that our in vitro model allows for identification of biomarkers that are useful for identifying cells which have undergone EMT and possibly those that are circulating tumor cells. Using this system to study cellular events involved in breast cancer development and progression would potentially lead to new diagnostic and therapeutic approaches for metastatic breast cancer.

## Supplementary Information

**Additional file 1**. Microarray image data from replicate 1 of BT-474 breast cancer cells exposed to fluid flow in a parallel-plate bioreactor system.

**Additional file 2**. Microarray probe-level data from replicate 1 of BT-474 breast cancer cells exposed to fluid flow in a parallel-plate bioreactor system.

**Additional file 3**. Microarray image data from replicate 2 of BT-474 breast cancer cells exposed to fluid flow in a parallel-plate bioreactor system.

**Additional file 4**. Microarray probe-level data from replicate 2 of BT-474 breast cancer cells exposed to fluid flow in a parallel-plate bioreactor system.

**Additional file 5**. Microarray image data from replicate 3 of BT-474 breast cancer cells exposed to fluid flow in a parallel-plate bioreactor system.

**Additional file 6**. Microarray probe-level data from replicate 3 of BT-474 breast cancer cells exposed to fluid flow in a parallel-plate bioreactor system.

**Additional file 7**. Microarray image data from replicate 1 of BT-474 breast cancer cells grown in static culture.

**Additional file 8**. Microarray probe-level data from replicate 1 of BT-474 breast cancer cells grown in static culture.

**Additional file 9**. Microarray image data from replicate 2 of BT-474 breast cancer cells grown in static culture.

**Additional file 10**. Microarray probe-level data from replicate 2 of BT-474 breast cancer cells grown in static culture.

**Additional file 11**. Microarray image data from replicate 3 of BT-474 breast cancer cells grown in static culture.

**Additional file 12**. Microarray probe-level data from replicate 3 of BT-474 breast cancer cells grown in static culture.

**Additional file 13**. Microarray image data from replicate 1 of MCF-7 breast cancer cells exposed to fluid flow in a parallel-plate bioreactor system.

**Additional file 14**. Microarray probe-level data from replicate 1 of MCF-7 breast cancer cells exposed to fluid flow in a parallel-plate bioreactor system.

**Additional file 15**. Microarray image data from replicate 2 of MCF-7 breast cancer cells exposed to fluid flow in a parallel-plate bioreactor system.

**Additional file 16**. Microarray probe-level data from replicate 2 of MCF-7 breast cancer cells exposed to fluid flow in a parallel-plate bioreactor system.

**Additional file 17**. Microarray image data from replicate 3 of MCF-7 breast cancer cells exposed to fluid flow in a parallel-plate bioreactor system.

**Additional file 18**. Microarray probe-level data from replicate 3 of MCF-7 breast cancer cells exposed to fluid flow in a parallel-plate bioreactor system.

**Additional file 19**. Microarray image data from replicate 1 of MCF-7 breast cancer cells grown in static culture.

**Additional file 20**. Microarray probe-level data from replicate 1 of MCF-7 breast cancer cells grown in static culture.

**Additional file 21**. Microarray image data from replicate 2 of MCF-7 breast cancer cells grown in static culture.

**Additional file 22**. Microarray probe-level data from replicate 2 of MCF-7 breast cancer cells grown in static culture.

**Additional file 23**. Microarray image data from replicate 3 of MCF-7 breast cancer cells grown in static culture.

**Additional file 24**. Microarray probe-level data from replicate 3 of MCF-7 breast cancer cells grown in static culture.

**Additional file 25**. Microarray image data from replicate 1 of MDA-MB-231 breast cancer cells exposed to fluid flow in a parallel-plate bioreactor system.

**Additional file 26**. Microarray probe-level data from replicate 1 of MDA-MB-231 breast cancer cells exposed to fluid flow in a parallel-plate bioreactor system.

**Additional file 27**. Microarray image data from replicate 2 of MDA-MB-231 breast cancer cells exposed to fluid flow in a parallel-plate bioreactor system.

**Additional file 28**. Microarray probe-level data from replicate 2 of MDA-MB-231 breast cancer cells exposed to fluid flow in a parallel-plate bioreactor system.

**Additional file 29**. Microarray image data from replicate 3 of MDA-MB-231 breast cancer cells exposed to fluid flow in a parallel-plate bioreactor system.

**Additional file 30**. Microarray probe-level data from replicate 3 of MDA-MB-231 breast cancer cells exposed to fluid flow in a parallel-plate bioreactor system.

**Additional file 31**. Microarray image data from replicate 1 of MDA-MB-231 breast cancer cells grown in static culture.

**Additional file 32**. Microarray probe-level data from replicate 1 of MDA-MB-231 breastcancer cells grown in static culture.

**Additional file 33**. Microarray image data from replicate 2 of MDA-MB-231 breast cancer cells grown in static culture.

**Additional file 34**. Microarray probe-level data from replicate 2 of MDA-MB-231 breast cancer cells grown in static culture.

**Additional file 35**. Microarray image data from replicate 3 of MDA-MB-231 breast cancer cells grown in static culture.

**Additional file 36.** Microarray probe-level data from replicate 3 of MDA-MB-231 breast cancer cells grown in static culture.

**Additional file 37**. Microarray image data from replicate 1 of SK-BR-3 breast cancer cells exposed to fluid flow in a parallel-plate bioreactor system.

**Additional file 38**. Microarray probe-level data from replicate 1 of SK-BR-3 breast cancer cells exposed to fluid flow in a parallel-plate bioreactor system.

**Additional file 39**. Microarray image data from replicate 2 of SK-BR-3 breast cancer cells exposed to fluid flow in a parallel-plate bioreactor system.

**Additional file 40**. Microarray probe-level data from replicate 2 of SK-BR-3 breast cancer cells exposed to fluid flow in a parallel-plate bioreactor system.

**Additional file 41**. Microarray image data from replicate 3 of SK-BR-3 breast cancer cells exposed to fluid flow in a parallel-plate bioreactor system.

**Additional file 42**. Microarray probe-level data from replicate 3 of SK-BR-3 breast cancer cells exposed to fluid flow in a parallel-plate bioreactor system.

**Additional file 43**. Microarray image data from replicate 1 of SK-BR-3 breast cancer cells grown in static culture.

**Additional file 44**. Microarray probe-level data from replicate 1 of SK-BR-3 breast cancer cells grown in static culture.

**Additional file 45**. Microarray image data from replicate 2 of SK-BR-3 breast cancer cells grown in static culture.

**Additional file 46**. Microarray probe-level data from replicate 2 of SK-BR-3 breast cancer cells grown in static culture.

**Additional file 47**. Microarray image data from replicate 3 of SK-BR-3 breast cancer cells grown in static culture.

**Additional file 48**. Microarray probe-level data from replicate 3 of SK-BR-3 breast cancer cells grown in static culture.

**Additional file 49**. Methodology for optimizing Smad2 and Smad3 siRNA transfections and for identifying ideal treatment times and efficiencies of transfections. Results of siRNA transfection optimization experiments and quantification of the effect of fluid flow on SNAI2 expression when flow is removed or when cells are exposed to lower fluid forces.

## Data Availability

The datasets analysed and presented in this manuscript are available from the corresponding author on reasonable request. All microarray datasets will be deposited in a public repository if the article is accepted for publication.
