# Supplementary material for: Fluid flow exposure promotes epithelial-to-mesenchymal transition and adhesion of breast cancer cells to endothelial cells
Source: Breast Cancer Res. 2021 Oct 12;23:97. doi: 10.1186/s13058-021-01473-0 (PMC8507133; doi:10.1186/s13058-021-01473-0)
Supplement: Supplementary file 49 — Additional file 49. Methodology for optimizing Smad2 and Smad3 siRNA transfections and for identifying ideal treatment times and efficiencies of transfections. Results of siRNA transfection optimization experiments and quantification of the effect of fluid flow on SNAI2 expression when flow is removed or when cells are exposed to lower fluid forces. [file 13058_2021_1473_MOESM49_ESM.docx]

**Fluid flow exposure promotes Epithelial-to-Mesenchymal Transition and adhesion of breast cancer cells to endothelial cells**

Kenneth F. Fuh^1^, Robert D. Shepherd^1,2^, Jessica S. Withell^1^, Brayden K. Kooistra^1^, Kristina D. Rinker^1,2,3,4,5^

^1^Cellular & Molecular Bioengineering Research Lab, ^2^Department of Chemical & Petroleum Engineering, ^3^Centre for Bioengineering Research & Education, ^4^Department of Physiology & Pharmacology, ^5^Libin Cardiovascular Institute of Canada; University of Calgary, Calgary, AB, Canada.

**Supplemental Methods and Results**

**Supplemental Methods**

*Optimization of Smad2 and Smad3 siRNA transfections:* Transfection experiments were optimized by seeding 2 wells of a 6 well cell culture plate with 200,000 MDA-MB-231 cells for each concentration of siRNA. Conditions included a no-treatment, control siRNA-A (sc-37007, Santa Cruz Biotechnology), and concentrations of 1, 5, 10, 25 and 50 nM of Smad2 or Smad3 siRNA. All transfections were performed for 4 h in 0.3% PepMute siRNA Transfection Reagent (SignaGen Laboratories, Rockville, MD, USA) in fresh media that had been changed 30 min beforehand. Afterwards, media was changed again, and the cells were left in static culture for 72 h (same time needed to set up flow experiments). RNA was harvested from samples and used for gene quantification and PCR experiments.

*Time course experiments:* Time course experiments were performed by seeding 2 wells of a 6 well cell culture plate with 200,000 MDA-MB-231 cells for 24 and 48 h. A fresh media change was made on cells 30 min before transfections. Cells were then transfected with 25 nM of Smad2 siRNA (Santa Cruz Biotechnology) for 4 h in 0.3% PepMute siRNA Transfection Reagent (SignaGen Laboratories, Rockville, MD, USA). Afterwards, a media change was made, and transfected cells were left in static culture until time of RNA harvest and subsequent PCR quantification.

*Transfection efficiency experiments:* Efficiency of transfections was determined by seeding a T-75 cell culture dish with 200,000 MDA-MB-231 breast cancer cells. After having grown for 24 h, the wells were transfected with 25 nM of BLOCK-iT™ Alexa Fluor® Red Fluorescent Control (Invitrogen) for 4 h in 0.3% PepMute siRNA Transfection Reagent (SignaGen Laboratories, Rockville, MD, USA). After transfection, cells were detached and re-seeded in 3 wells of a 6 well plate and left to grown for 24 h. Cells were then washed twice in PBS and fixed in 4% PFA (Acros Organics-Thermo Fisher Scientific, Ottawa, ON, Canada) for 10 mins before being washed again twice in PBS. Cells were imaged under the Olympus FV1000 confocal microscope (Olympus Inc., Waltham, MA, USA) with a 10x objective and whole well pictures were taken. Cells in three random microscopic fields were individually counted in each well and determined transfected if a noticeable amount of positive red signal had shown in the cell. Three independent experiments were performed at each time point.

**Supplemental Results**

**A B**

**
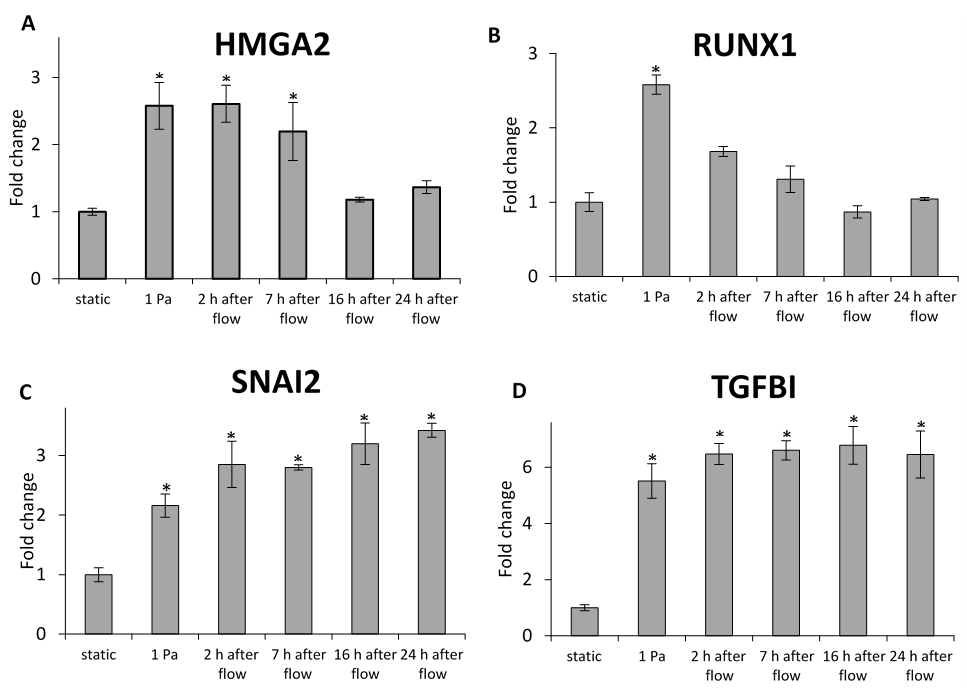
**
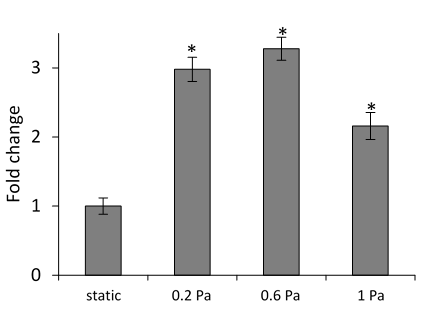


**Supplemental Fig. 1:** **(A)** Quantitative RT-PCR analysis of SNAI2 expression in MDA-MB-231 cells stimulated with fluid flow for 20 h at 1 Pa, then left in static conditions for 2, 7, 16 and 24 h. **(B)** Quantitative RT-PCR analysis of SNAI2 expression in MDA-MB-231 cells stimulated with fluid flow for 20 h at 0.2, 0.6 and 1 Pa. Fold changes were calculated using the comparative cycle threshold method and normalized to expression in static conditions. Statistical significance threshold was set at fold change ≥ 2 and p < 0.05. Asterisks indicate statistically significant fold changes in flow or post flow stimulated cells with respect to expression in unstimulated (static) cells. Statistical significance was determined using the Student’s t-test. Duplicate wells were run for every sample. B2M was used as the reference gene. Data shown represent the means ± standard errors of the means of data from at least 3 samples for each condition.

***Smad2 and Smad3 siRNA optimization experiments***

To investigate involvement of Smad2 and Smad3 in transducing the mechanical stimulus from fluid flow to upregulate HMGA2, RUNX1, TGFBI and SNAI2 in breast cancer cells, Smad2 and Smad3 were knocked down using corresponding small interfering RNA (siRNA). Abolition of flow effects on expression of these genes were monitored after knockdown experiments. First, several experiments were performed to optimize and validate effective knockdown by siRNAs. These included optimizing concentration of each siRNA, negative and endogenous positive controls, and transfection efficiency experiments.

*Optimizing siRNA concentration:* To determine optimal siRNA concentrations required for effective Smad2 and Smad3 knock downs, MDA-MB-231 cells were transfected with no siRNA, Control siRNA, 1, 5, 10, 25 and 50 nM Smad2 or Smad3 siRNA. RNA was harvested from the cells and used to quantify gene expression using PCR. These results, shown in Supplemental Fig. 2, suggested a siRNA concentration of 25 nM would provide a strong knock down effect for both Smad2 and Smad3. At this concentration, expression of Smad2 and Smad3 were respectively knocked down by 91% and 86% using corresponding siRNAs, while also maintaining cell integrity (Supplemental Table 1).


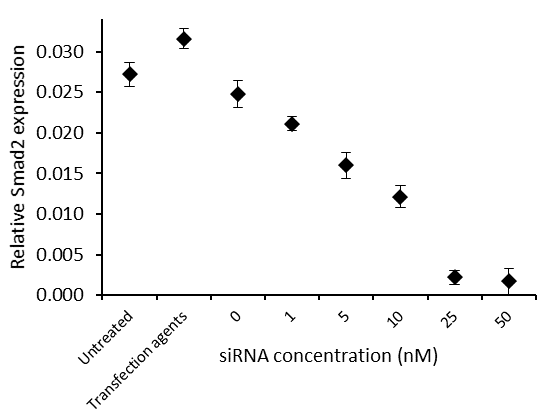

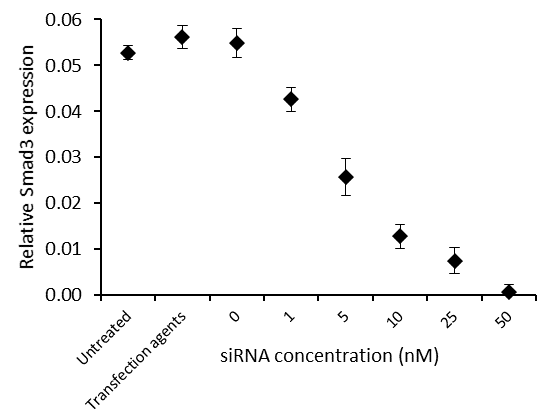


**A**

**B**

**Supplemental Fig. 2:** Relative expression of Smad2 (A) and Smad3 (B) genes after transfections with no siRNA (untreated control), transfection agents only (without any siRNA), 0 (Control siRNA), 1, 5, 10, 25 and 50 nM siRNA. Data shown represent the means ± standard errors of the means of data from at least 3 samples for each condition. Duplicate wells were run for every sample. B2M was used as the reference gene.

*Endogenous positive control experiments:* To determine whether or not the transfection reagents affected expression of Smad2 or Smad3, endogenous positive control experiments were performed in which MDA-MB-231 cells were treated with transfection media consisting all transfection reagents except the siRNA. Expression of Smad2 and Smad3 were quantified and compared to expression in non-transfected cells. Supplemental Fig. 2 shows similar Smad2 and Smad3 mRNA levels in cells transfected with the reagents alone and in non-transfected (untreated) cells, indicating that the transfection reagents had no effect on expression of both Smad2 and Smad3.

**Supplemental Table 1:** Calculation of percent knockdown of Smad2 and Smad3 in MDA-MB-231 breast cancer cells. Relative expression of Smad2 and Smad3 were quantified in cells transfected with control siRNA or corresponding siRNA (25 nM). Data shown represents the mean from 3 samples for each condition. Gene expression was quantified using qPCR. Duplicate wells were run for every sample. B2M was used as the reference gene.

|  | **Average relative expression** | | **% knockdown** |
| --- | --- | --- | --- |
|  | control siRNA | siRNA (25 nM) |  |
| **Smad2** | 0.0248 | 0.0022 | 91 |
| **Smad3** | 0.0549 | 0.0075 | 86 |

*siRNA negative control experiment:* To distinguish sequence-specific silencing from non-specific siRNA downstream effects, MDA-MB-231 cells were transfected with a siRNA negative control consisting of a scrambled sequence. Samples were analyzed similarly as in other transfection experiments. Supplemental Fig. 2 shows no significant differences in relative expression of Smad2 or Smad3 between cells transfected with control siRNA (0 nM of siRNA) and non-transfected cells, indicating that silencing by Smad2 or Smad3 siRNA is specific, and any downstream effects to gene knockdown can be attributed to the specific silencing of the target genes.

*Transfection Efficiency:* To determine efficiency of transfection experiments, MDA-MB-231 cells were transfected with BLOCK-iT™ Alexa Fluor® Red Fluorescent Control and extent of red dye uptake analyzed 24 and 48 h post transfection. This was done by individually counting cells that had a noticeable amount of positive red signal per total number of cells in a particular microscopic field. Supplemental Fig. 3 shows percent transfection efficiencies at each time point. These results indicate that for the MDA-MB-231 cells used in transfection experiments, siRNA uptake is achieved and maintained in more than 80% of cells by the time RNA is harvested from transfected cells following flow exposure (48 h).

Supplemental Fig. 3: Percent transfection efficiencies at 24 and 48 h post transfection with the Alexa Fluor® Red Fluorescent Oligo Control. Transfection efficiency was calculated as percent of cells with the oligo control per total number of cells in a particular microscope field. Each well was seeded with 200,000 cells. Cells in 3 random microscopic fields were counted for each group. The results presented are an average of 3 random microscopic fields from 3 independent experiments. Data shown represent the means ± standard errors of the means of data from 3 independent experiments.
